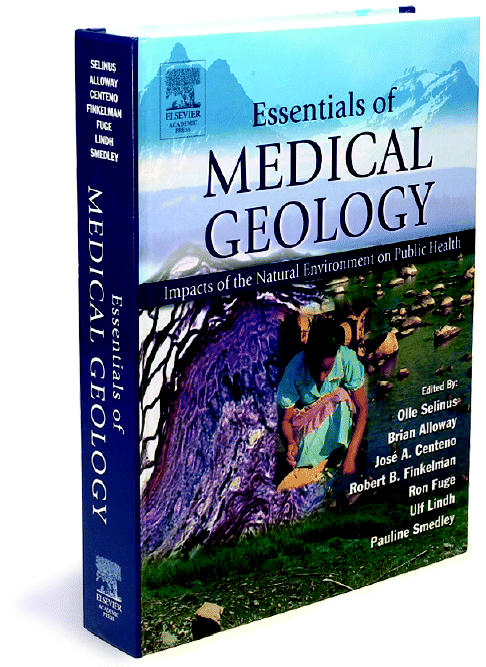# Essentials of Medical Geology: Impacts of the Natural Environment on Public Health

**Published:** 2005-11

**Authors:** Mark Pokras

**Affiliations:** Mark Pokras trained in ecology at Cornell University and earned his DVM at Tufts University’s Cummings School of Veterinary Medicine, where he currently teaches wildlife medicine and serves as Director of Tufts’ Center for Conservation Medicine.

Edited by Olle Selinus, Brian Alloway, José A. Centeno, Robert B. Finkleman, Ron Fuge, Ulf Lindh, and Pauline Smedley

Burlington, MA:Elsevier Academic Press, 2005. 812 pp. ISBN: 0-1263-6341-2, $99.95 cloth

Emerging disease, pesticides, antibiotic resistance, heavy metals—every time we turn around it seems we face frightening new threats to the health of every living organism on our planet. In response, we have seen a dramatic increase in the development of new, transdisciplinary approaches including environmental medicine, conservation medicine, health social science, and One World, One Health—but medical geology?

Readers will not have to get far into this book to become convinced that geologic expertise has much to contribute to our understanding of and response to global health issues. Medical geology, which examines the impacts of geologic materials and processes on human and ecosystem health including both natural and anthropogenic sources of potential health problems, includes animal and plant diseases. The editors set ambitious goals for this book, noting in the preface that this volume could be used as both a reference and a general textbook for a diverse audience including students, geoscientists, medics, decision makers, and the general public.

The first section, “Environmental Biology,” builds from individual inorganic reactions to cells, organisms, and ecosystems, laying a sound foundation for the concepts to follow. For those with medical backgrounds, one of the most useful aspects is the very different view that geologists bring to health issues. This section builds a firm foundation for the subsequent chapters, intertwining geologic and biologic chapters.

The second section, “Pathways and Exposures,” focuses primarily on “natural sources” of pollutants and their transport through air, water, and food chains and demonstrates the importance of unifying and integrating themes for understanding long-term, large-scale processes in ecosystem dynamics. Ecologic concepts are integrated with the epidemiologic and illustrated by real-world examples and experiments. Most chapters are fundamentally strong, but biologists may wish for deeper discussions of topics such as biologic magnification and effects on predatory species.

The third section, “Environmental Toxicology, Pathways and Medical Geology,” includes a significant focus on epidemiology. This section is strong but repeats many basic principles (e.g., the metabolic handling of exogenous chemicals) and specific examples (e.g., discussions of arsenic, mercury, and lead) discussed in early chapters. The presence of contrasting explanations, opinions, and viewpoints can serve important didactic functions. Given that medically oriented authors wrote most of these chapters, this section may hold the most exciting ideas for the geologic readership.

The last section, “Techniques and Tools,” is an excellent reminder of the breadth of applications included in medical geology. The discussions span imaging techniques from cellular to global and analytic methodologies from the molecular to tissue, watershed, and continental scales. Necessarily, many abstruse or cutting-edge techniques have not been included, but the medical audience will find relevance in those that are focused upon.

This wide-ranging and challenging introduction is filled with wonderful and frightening examples from around the world. The amalgam of theoretical, ecologic, and clinical cases with discussions of policy is one of the book’s strongest points. The focus is primarily on human health, and although some examples involve domestic animals or plants, almost none address nonmammalian (especially nonvertebrate) species. Some chapters assume a fair degree of quantitative sophistication, so instructors should ensure that the text matches student abilities. Much of the material will excite students and involve them in analysis and discussion. Most chapters include a useful summary or conclusions, a list of related topics in other chapters, and a list of further reading, and many chapters emphasize a significant problem faced by researchers and policy makers working on the natural world.

This tour de force has several great strengths, including the marvelously international contributors. More than a theoretical approach, this volume is packed with real-world examples, cases, and information from research, clinical, and policy perspectives. Excellent illustrations, graphs, and photographs also complement many chapters and add markedly to the value of the book. Unfortunately, repetition of key examples throughout the text has made the book a bit less useful than some readers might wish; more diversity might be desirable in later editions.

For health specialists, graduate students, and the technically inclined, this book will be an invaluable resource. But it is a bit too large, technical, and imposing to be called “Essentials.” The editors might consider preparing an abbreviated introductory text to attract a wider audience. The book is a forceful reminder that we need more geologic input incorporated into health assessments, environmental toxicology studies, and planning and policy initiatives—as recent events on the Gulf Coast of the United States so strongly demonstrate.

## Figures and Tables

**Figure:**